# Can the Large-Scale Decrement in Repetitive Nerve Stimulation Be Used as an Exclusion Criterion for Amyotrophic Lateral Sclerosis?

**DOI:** 10.3389/fneur.2020.00101

**Published:** 2020-02-28

**Authors:** Li Shang, Hong Chu, Zuneng Lu

**Affiliations:** Department of Neurology, Renmin Hospital, Wuhan University, Wuhan, China

**Keywords:** amyotrophic lateral sclerosis, repetitive nerve stimulation, electrophysiological diagnostic criteria, compound muscle action potential, neuromuscular junctions

## Abstract

**Objective:** The objectives of this work were to identify the characteristics of repetitive nerve stimulation (RNS) in patients with amyotrophic lateral sclerosis (ALS) and further verify the electrophysiological exclusion criteria of ALS.

**Methods:** A total of 150 patients with ALS who were admitted to the Department of Neurology of Renmin Hospital of Wuhan University from January 2015 to December 2018 were enrolled. Clinical and electrophysiological data of the enrolled patients were collected. The differences in the amplitudes of the compound muscle action potential (CMAP) between the trapezius muscle (Trap) and the abductor digiti minimi (ADM) in low-frequency RNS were compared. Furthermore, we analyzed the associations between decremental responses and gender, onset age, duration of disease, onset site, Amyotrophic Lateral Sclerosis Functional Rating Scale—Revised (ALSFRS-R), disease progression rate, and CMAP amplitude.

**Results:** A significant decrement (≥20%) in at least one muscle was observed in 11.3% of the ALS patients, while decrements (≥10%) in at least one muscle were observed in 41.3%. The decremental percentage in the trapezius muscle was significantly higher than that in the abductor digiti minimi (*P* < 0.001). The onset age, duration of disease, onset site, and disease progression rate did not affect decremental responses. The decremental responses in RNS were more significant in ALS patients with low ALSFRS-R scores (*P* = 0.01). Moreover, there was a positive linear correlation between the CMAP amplitude and the decremental percentage of Trap and ADM in ALS patients.

**Conclusions:** CMAP decremental responses in RNS were common in ALS patients, suggesting abnormalities of neuromuscular junctions (NMJs). It is worthy of further discussion whether to consider a decrement >20% in RNS as a diagnostic exclusion criterion for ALS.

## Introduction

Amyotrophic lateral sclerosis (ALS), the most quintessential motor neuron disease, affects both upper and lower motor neurons, and leads to progressive muscle atrophy and weakness (1). Mulder et al. (2) originally reported that decremental responses in low-frequency repetitive nerve stimulation (RNS) tests were observed in ALS patients. Subsequently, domestic and foreign studies have confirmed this phenomenon (3–5). The electrophysiological diagnostic criteria of ALS revised by the World Federation of Neurology Research Group on Motor Neuron Diseases pointed out that a significant compound muscle action potential (CMAP) decrement >20% in RNS was a diagnostic exclusion criterion for ALS (6). However, later studies demonstrated that a decrement >20% was observed in ALS patients (4, 7, 8). So far, none of the published studies has indicated the proportion of patients with a decrement of 20% or greater. And the exclusion criteria have not been extensively validated. In addition, the amplitude and incidence of decremental responses and the criteria for the positive decrement were inconsistent among previous research.

In this study, we described the amplitude and incidence of CMAP in low-frequency RNS of ALS patients and explored the correlations between decremental responses and clinical characteristics. We further validated the electrophysiological diagnostic exclusion criteria of ALS, which will enable a better understanding of the disease diagnosis.

## Methods

### Patients

A total of 150 patients at the Department of Neurology of Renmin Hospital of Wuhan University were recruited from January 2015 to December 2018. According to the Awaji criteria (9), patients were classified into probable ALS (43 patients) and definite ALS (107 patients). All patients were subjected to rigorous neurological examinations, EMG examinations, blood tests, including serum acetylcholine (ACH) receptor antibodies, cerebrospinal fluid examinations, and imaging studies to exclude important ALS mimics. The exclusion criteria were as follows: (1) patients with a history of poliomyelitis; (2) patients with a positive response to ACH receptor antibodies; (3) patients with spinal cord tumor and with autoimmune disorders; (4) patients with other diseases affecting peripheral nerves, neuromuscular junction (NMJ), or muscles; and (5) patients without complete clinical records.

### Method of RNS

All patients underwent routine electromyography performed on a Keypoint Workstation (31A06, Alpine BioMedApS, Denmark), including nerve conduction tests, needle electromyography, and RNS. The detection method followed the national guidelines (10). The surface electrode was stimulated and recorded, and the response to the stimulus was recorded 10 times each time. The accessory nerve was stimulated on the posterior border of the sternocleidomastoid. The ulnar nerve was stimulated on the wrist. The surface recording electrodes were used to record over the belly of the trapezius muscle (Trap) and the abductor digiti minimi (ADM), which respectively represented the proximal muscle and the distal muscle. The reference electrodes were placed on the tendon of the tested muscle. The skin temperature over the examined muscle was maintained at 33°C or above throughout the examination. Filters were set between 20 Hz and 3 kHz.

### Study Design

The Amyotrophic Lateral Sclerosis Functional Rating Scale—Revised (ALSFRS-R) was used to calculate the monthly rate of change in ALSFRS-R, which represented the disease progression rate. The greater the value of progression obtained, the faster the disease progressed:

$$\begin{array}{l} Disease\;progression\;rate\\ \;=\left(48\;-\;actual\;score\right)/duration\;\left(months\right) \end{array}$$

The decremental responses at a frequency of 3 Hz were mainly analyzed. The baseline-to-negative peak amplitudes of the first and fifth CMAPs at the supramaximal intensity (1.25-fold maximal intensity for the M-wave response) were measured. The CMAP decrement in the peak-to-peak amplitude was measured by the decremental percentage of the fifth CMAP as compared to the first CAMP amplitude. Decremental responses of ≥10% were defined as positive. Patients were divided into the positive decrement group and the negative decrement group based on whether the decrement was ≥10%. The data on the lower side of the CMAP amplitude was the standard. The correlations between the decremental responses and ALSFRS-R scores and CMAP amplitude were analyzed.

### Statistical Analysis

Statistics calculations were performed on the SPSS 22.0 software. The Kolmogorov–Smirnov test was used to evaluate whether the data were normally distributed. Parameters with a normal distribution were described as the mean ± standard deviation (SD), and those with a non-normal distribution were expressed as the median values (*M*) and interquartile range (*Q*). Continuous variables that were normally distributed were compared using Student's *t*-test. Mann–Whitney *U*-tests were implemented to analyze values with a non-normal distribution. The categorical variables were expressed as the number of cases (*n*) and percentage (%), and the chi-squared test was used for comparison between groups. Correlation analysis was performed using Spearman's test. *P* < 0.05 was considered to be significant.

## Results

### General Information

There were 85 males and 65 females, with ages from 28 to 80 years (mean age of 56.66 ± 11.29 years), in the included patients. The median duration from symptom onset was 14.34 ± 12.69 months. The ALSFRS-R scores were in the range 24–46, and the median was 40.5. There were 75 cases with upper limb onset and 41 cases with lower limb onset. The rest of the cases were with bulbar onset.

### RNS Results

**Figure 1:**
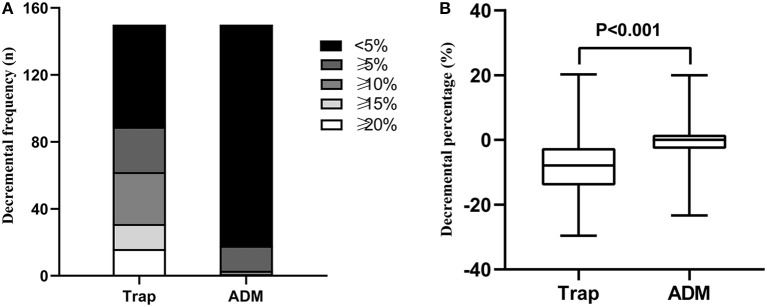
**(A)** Decremental frequency in the Trap and ADM of ALS patients. **(B)** Decremental percentage in the Trap and ADM of ALS patients. Trap, trapezius muscle; ADM, abductor digiti minimi.

The distributions of decremental responses are summarized in Table 1. When the CMAP decrement of ≥10% was used as a criterion, the positive decrement in at least one muscle accounted for 41.3% (62/150), most of which were in the proximal muscle (Figure 1). The CMAP decremental percentage ranged from 10 to 29.57%, with an average of 16.63% in patients with a positive decrement.

**Table 1:** RNS decremental amplitude and frequency in ALS patients.

**Decremental amplitude**	**Trap**		**ADM**	
	***n***	**%**	***n***	**%**
≥20%	16	10.7	1	0.7
≥15%	15	10.0	0	0.0
≥10%	31	20.7	2	1.3
≥5%	27	18.0	15	10.0
<5%	61	40.6	132	88.0
Total	150	100.0	150	100.0

*Trap, trapezius muscle; ADM, abductor digiti minimi; RNS, repetitive nerve stimulation; ALS, amyotrophic lateral sclerosis*.

### Correlation Between Decremental Response and Clinical Features and CMAP Amplitude

**Figure 2:**
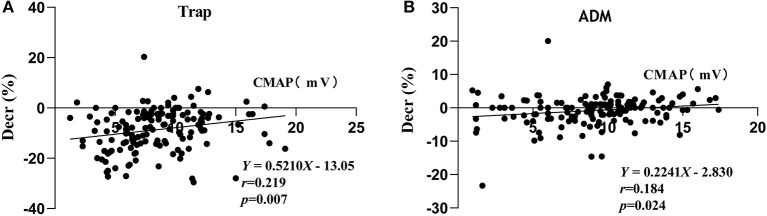
Correlations between the CMAP amplitude and decremental percentages in TRAP **(A)** and ADM **(B)**. Decr(%), percentage of decremental response; CMAP, compound muscle action potential; Trap, trapezius muscle; ADM, abductor digiti minimi.

Compared with the patients in the negative decrement group, the patients in the positive decrement group showed lower ALSFRS-R scores and CMAP amplitudes in both proximal and distal muscles (Table 2). There was a positive linear correlation between the CMAP amplitude and the decremental percentage in Trap and ADM of ALS patients (*r* = 0.219, *P* = 0.007; *r* = 0.184, *P* = 0.024, respectively; Figure 2).

**Table 2:** Comparison of positive and negative decrements in ALS patients.

	**RNS+**	**RNS–**	***t*/*z*/χ^**2**^**	***P***
Sex, *n* (%)				
Male	32 (51.6)	54 (61.4)	1.414^*^	0.234
Female	30 (48.4)	34 (38.6)		
Onset age (years)	55.12 ± 11.95	55.71 ± 10.63	−0.316^†^	0.752
Disease duration (months) [*M*(*Q*)]	12.00 (11.5)	9.50 (11.5)	−1.695^§^	0.090
ALSFRS-R [*M*(*Q*)]	39.00 (6.0)	41.00 (5.0)	−2.588^§^	0.010
Disease progression rate [*M*(*Q*)]	0.75 (0.7)	0.70 (1.0)	−0.220^§^	0.826
Onset site, *n* (%)				
Bulbar	12 (19.4)	22 (25.0)	0.661^*^^,^ ^ϕ^	0.416
Upper limb	30 (48.4)	45 (51.1)	0.833^*^^,^ ^&^	0.316
Lower limb	20 (32.3)	21 (23.9)		
CMAP amplitude (mV)				
Trap	7.53 ± 3.59	9.10 ± 3.25	−2.788^†^	0.006
ADM	7.99 ± 3.74	9.94 ± 3.39	−3.319^†^	0.001

+, Positive decrement; –, Negative decrement.

ALS, amyotrophic lateral sclerosis; CMAP, compound muscle action potential; Trap, trapezius muscle; ADM, abductor digiti minimi.

* χ^2^-value.

§ z-value.

† t-value.

ϕ Bulbar vs. limb onset.

& *Upper limb onset vs. lower limb onset*.

## Discussion

A number of studies have demonstrated that decremental responses were frequently observed in ALS patients. Nevertheless, the positive rate of decremental responses varied due to the diverse sample sizes, muscles, and decremental positive criteria. When the CMAP decrement of ≥10% was defined as abnormal, the positive rate of decremental responses was 77% in one study with 48 ALS patients (5), while that in another study including 192 patients in total was only 29% (3). The positive rate of the present study was 41.3%. All of these indicated that decremental positive responses in RNS were common in ALS patients.

In light of the revised ALS diagnostic criteria by the World Federation of Neurology (6), it is noteworthy that a CMAP decrement >20% was a diagnostic exclusion criterion for ALS. However, our study investigated that more than 20% of the patients had a decrement of ≥15%. Furthermore, more than 10% of the patients had a decrement of ≥20%. Although it has also been reported previously that the decremental percentage can reach 20% (4, 7, 8), the proportion of patients with such a characteristic was not mentioned. If the current electrophysiological diagnostic criteria were applied, more than 10% of ALS patients would be misdiagnosed. Consequently, it is worthy of further research whether a CMAP decrement >20% is an exclusion criterion for ALS.

The present study analyzed the distribution of the decremental responses in Trap and ADM. Consistent with previous research (3, 5), the investigation demonstrated that proximal muscles were more prone to show a positive decrement in RNS than do distal muscles, indicating a greater sensitivity of the proximal muscle to RNS. In severely atrophied muscles, the decremental responses of ALS patients were more pronounced (3, 11); however, it cannot be explained why more severely involved distal muscles, such as intrinsic hand muscles, in most ALS patients showed a less pronounced decremental positive response than did the proximal muscles. The lower safety factor of NMJ at the proximal muscle may be an interpretation (5). Besides, the study indicated that there was a positive linear correlation between the CMAP amplitude and the decremental percentage of Trap and ADM in ALS patients, which supported the decremental mechanisms of immature collateral sprouting and unstable NMJ transmission resulting from axonal degeneration. The Awaji diagnostic criteria indicated that electrophysiological and clinical evidence should be considered equally when judging the lower motor neuron (LMN) involvement in ALS patients (9). The decremental positive responses in RNS meant an impaired LMN (12). Accordingly, when the Awaji diagnostic criteria were applied, the decremental positive responses can be deemed as an additional evidence of the LMN involvement (12).

The ALSFRS-R score, as a significant predictor of prognosis, was extensively applied to assess the severity of the disease (7). The patients in the positive decrement group had lower ALSFRS-R scores compared with those in the decremental negative group, suggesting that the former were worse. Some studies have shown that the disease progressed more rapidly in ALS patients with a positive decrement (4, 8, 13, 14), but others contradicted this (5, 7, 15–17). If the RNS decrement responses were not related to the disease progression, the interpretation of the mechanism of decrement responses, the early neural reinnervation, may need to be corrected (5). It may be possible that the disease progression was too rapid to form the collateral sprouts in extreme cases. Therefore, the correlation between the RNS decremental responses and the disease progression rate in ALS patients is still confused. Consequently, advanced research is essential to clarify that the decremental positive responses are monitoring indicators of disease progression.

Moreover, this study showed that the onset of ALS did not affect the RNS decrement responses, which was consistent with previous research (8, 18). However, some studies have shown that patients with upper limb onset had higher decremental percentages compared with those with other sites of onset (7, 17). The underlying reason for these differences is unclear. It was speculated that the limited number of patients with lower limb and bulbar onset as well as the different types of muscles studied may be the underlying interpretations. In any case, more specifically designed research would be necessary to settle this issue. Besides, the patients' gender, onset age, and disease duration did not affect the RNS decremental percentages.

The decremental responses of ALS patients suggest the instability of NMJ transmission. The underlying mechanism of decremental responses remains controversial. One explanation is that, as the disease progresses, the anterior horn motor neurons of the spinal cord degenerate; then, the muscle fibers dominated by these neurons show denervation, leading to the corresponding NMJ structure changes. The remaining motor neurons around the degenerative neurons dominate the muscle fibers by the collateral sprouts, and new NMJ structures form. Before the new NMJ structures are fully mature, the motor neurons have degenerated and lost. Along with the repeated cycles, these highly immature reinnervations cause an intermittent conduction block in the newly formed nerve endings, eventually leading to a decremental response (4, 19). Another possible explanation is that the disease arises from distal axons or NMJs and proceeds to the cell body in reverse. This is the “dying back” hypothesis (17). Fischer et al. performed a good deal of pathological experimentation on the SOD1-G93A mice model and found that NMJ destruction and axon degeneration occurred before motor neurons degenerated. An autopsy on a single ALS patient showed denervation and reinnervation changes in muscles, where the motor neurons remained structurally intact (20). The mechanism of “dying back” remains unclear. A possible explanation is energy deficiency resulting from the hypermetabolism and malnutrition of ALS, which could lead to NMJ dysfunction (17).

In conclusion, the decremental positive responses are common in ALS patients, but the pathophysiological mechanisms remain unclear. Advanced research is necessary to discuss whether the decremental percentage >20% is a diagnostic exclusion criterion for ALS patients. Patients with lower CMAP amplitudes were more prone to show decremental responses, both in the proximal and distal muscles. Besides, the onset age, duration of disease, and onset site of ALS do not affect RNS the decremental responses.

## Data Availability Statement

All datasets generated for this study are included in the article/supplementary material.

## Ethics Statement

Ethics approval and written informed consent was not required as per local legislation and national guidelines, as repetitive nerve stimulation inspection is considered routine care.

## Author Contributions

LS drafted the paper. HC was responsible for data collection. ZL provided specialized expertise and critical appraisal of the article for submission.

### Conflict of Interest

The authors declare that the research was conducted in the absence of any commercial or financial relationships that could be construed as a potential conflict of interest.
